# Magnetic Resonance Imaging-Negative Varicella Zoster Virus Plexopathy in a Young Patient: A Case Report

**DOI:** 10.7759/cureus.39876

**Published:** 2023-06-02

**Authors:** Sedat Gül, Adeenah F Ahmed, Corey McGraw, Ruham Alshiekh Nasany

**Affiliations:** 1 Neurology, State University of New York Upstate Medical University, Syracuse, USA

**Keywords:** shingles, lomustine, procarbazine, varicella-zoster virus, brachial plexopathy

## Abstract

Varicella zoster virus (VZV)-associated plexopathy mainly occurs in patients over 60 years old. Postherpetic neuralgia is a well-known complication of herpes zoster (HZ); however, segmental zoster paresis secondary to HZ was reported in 1-20% of cases in the literature. Magnetic resonance imaging (MRI) findings may be positive in up to 70% of the patients. We describe a 43-year-old male patient with a history of grade two left frontal oligodendroglioma, which was treated with two partial resections, radiation treatment and procarbazine/lomustine, who presented with left upper extremity pain and developed a blistering rash in a dermatomal pattern in the left proximal upper extremity two weeks after the initial symptoms. He was diagnosed with shingles and treated with steroids and acyclovir with minimal improvement. Six weeks after the initial symptoms, a physical exam revealed left deltoid, supraspinatus and infraspinatus weakness with normal muscle stretch reflexes and decreased sensation on the C5 dermatome. Electromyography (EMG) revealed absent left lateral antebrachial cutaneous sensory nerve action potentials (SNAP) amplitude and a small left radial SNAP amplitude compared to the right side. Evidence of ongoing denervation with reinnervation was seen in the left upper trunk-supplied muscles. MRI of the brachial plexus was negative for any abnormalities. The patient was diagnosed with VZV-associated plexopathy, which improved with pregabalin and physical therapy. Our patient was significantly younger than expected in the HZ group. MRI usually shows T2 hyperintensities and thickening of the nerve roots in patients with VZV-associated plexopathy. However, the presentation, onset of symptoms, characteristics of the rash, and clinical course were diagnostic of HZ, and the weakness pattern, supported by the EMG findings, was diagnostic of VZV-associated plexopathy.

## Introduction

Varicella zoster virus (VZV) is a DNA virus capable of establishing latency within the sensory ganglia. If reactivated later in life or secondary to an immunocompromised state, the clinical condition is known as herpes zoster (HZ), or colloquially, shingles. Shingles is known to mainly affect the elderly, with a majority of cases occurring in those 50 years and older [1]. Its presentation involves a characteristic painful, vesicular rash along a specific dermatome unilaterally, making its diagnosis routinely clinical. Patients often experience a prodromal period of pain and paresthesia along the innervation of the affected sensory nerve for two or more days before vesicles erupt [2]. The vesicles progress into pustules and then scabs, taking roughly a week or two before the skin begins to heal [2]. Treatment involves antiviral therapy such as acyclovir, which is most effective if administered within 72 hours of the onset of a rash [3]. Early intervention is also critical in preventing complications post-infection. Also utilized in the management of HZ is pain relief as needed with medications such as gabapentin, an anticonvulsant. While postherpetic neuralgia is a well-known neurological complication, there are other sometimes overlooked complications too, such as motor neuropathy. Current literature notes that segmental zoster paresis secondary to HZ has been noted in merely 1% to 20% of cases [4-6].

Brachial plexopathy occurs with traumatic or nontraumatic insults to the brachial plexus, resulting in motor or sensory symptoms of the arm and shoulder consistent with peripheral neuropathy. Causes can include direct injuries like trauma from birth, exposure to toxins, chemicals, or drugs, and inflammatory conditions such as autoimmune disorders or infectious processes like viral illnesses, including HZ [7]. Electromyography (EMG) is utilized to locate, evaluate the severity of, and determine recovery prospects in cases of brachial plexopathy [7].

Here we describe a case of MRI-negative HZ brachial plexopathy, confirmed by EMG, in a 44-year-old patient with a past medical history significant for oligodendroglioma status post-chemotherapy and radiotherapy.

## Case presentation

**Figure 1:**
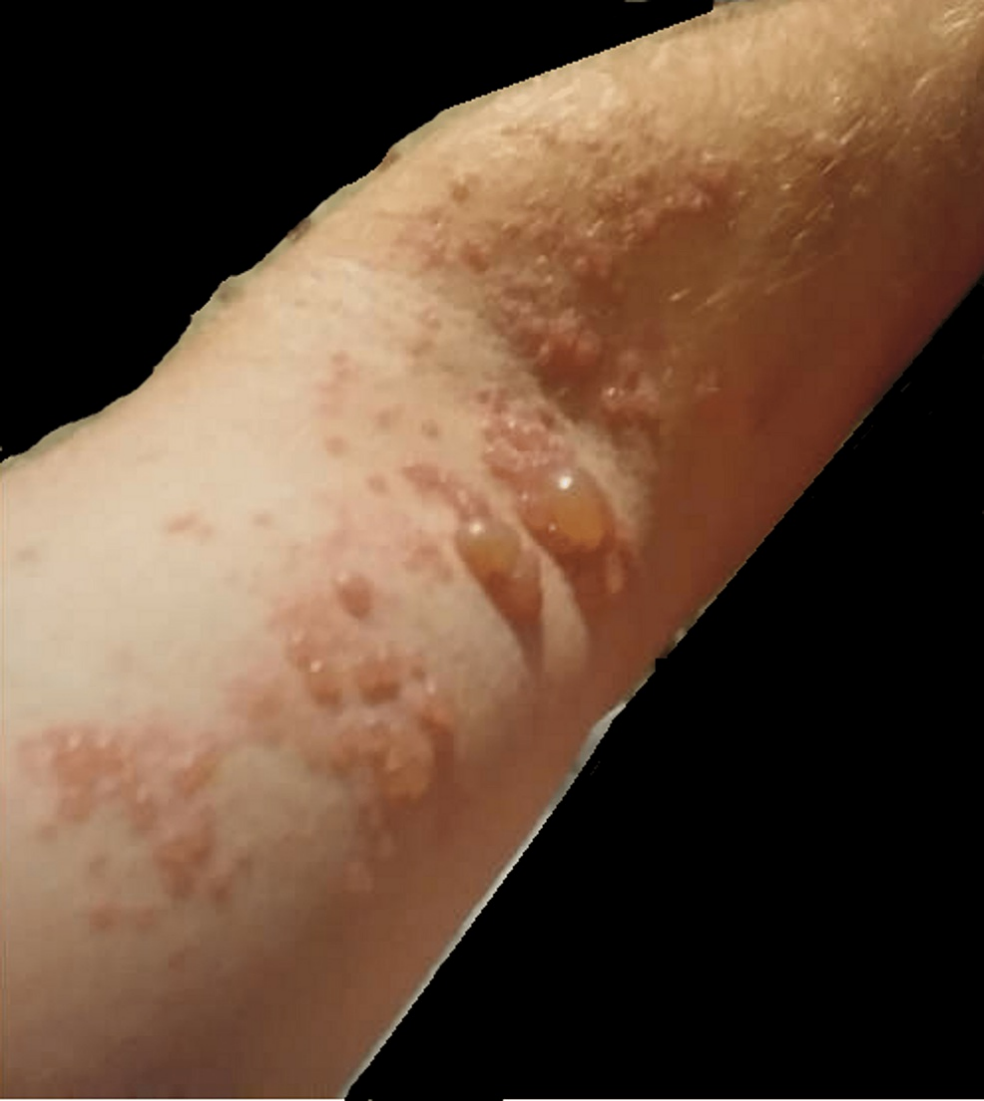
Blistering rash on the left upper extremity

A 43-year-old male presented with shoulder pain and weakness to the clinic. His past medical history was significant for grade 2 left frontal oligodendroglioma. He had two partial resections, radiation treatment, and procarbazine/lomustine for five cycles, which was completed two years prior to his presentation. He was put on levetiracetam 750 mg twice a day for seizure prophylaxis. He started experiencing weakness and pain in his left upper extremity and developed a painful and blistering rash around his left shoulder two weeks after the onset of symptoms (Figure 1).

**Figure 2:**
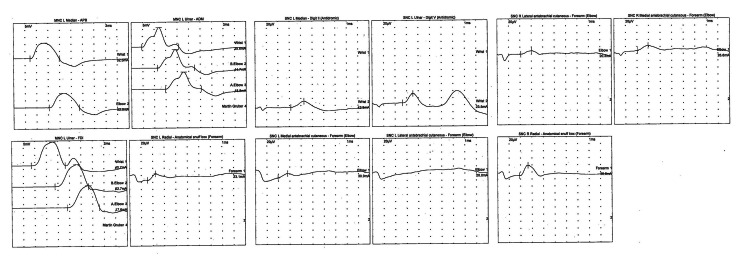
Electromyography waveforms

He was subsequently admitted to the hospital for shingles, and treated with steroids and acyclovir. Pain was treated with gabapentin and hydrocodone with only minimal improvement even after a month of treatment with these medications. By then, the patient had been experiencing pain, weakness, and numbness for approximately two months. A physical exam revealed atrophy of the left deltoid, supraspinatus and infraspinatus muscles, with muscle strength of only 2/5. He was unable to perform abduction above 10 degrees. Muscle stretch reflexes were symmetrical without any hyperreflexia. Sensation was decreased in the dermatome innervated by the C5 root. An EMG revealed absent left lateral antebrachial cutaneous sensory nerve action potentials (SNAP) amplitude, and a small left radial SNAP amplitude compared to the right side (Figure 2, Table 1).

**Table 1:** Electromyography depicting an absent left lateral antebrachial cutaneous sensory nerve action potentials (SNAP) amplitude and a small left radial SNAP amplitude compared to the patient’s right side

Nerve / sites	Rec. Site	Onset Lat ms	Peak Lat ms	NP Amp uV	Segments	Distance mm	Velocity m/s
L Median – Digit II (Antidromic)							
Wrist	Dig II	3.13	4.27	17.8	Wrist – Dig II	140	45
L Ulnar – Digit V (Antidromic)							
Wrist	Dig V	2.66	3.54	26.4	Wrist – Dig V	140	53
L Radial – Anatomical snuff box (Forearm)							
Forearm	Wrist	1.56	2.24	18.3	Forearm – Wrist	100	64
R Radial – Anatomical snuff box (Forearm)							
Forearm	Wrist	1.93	2.66	29.4	Forearm – Wrist	100	52
L Lateral antebrachial cutaneous – Forearm (Elbow)							
Elbow	Forearm	NR	NR	NR	Elbow – Forearm	120	NR
R Lateral antebrachial cutaneous – Forearm (Elbow)							
Elbow	Forearm	2.08	3.02	9.5	Elbow – Forearm	120	58
L Medial antebrachial cutaneous – Forearm (Elbow)							
Elbow	Forearm	1.98	2.81	12.4	Elbow – Forearm	120	61
R Medial antebrachial cutaneous – Forearm (Elbow)							
Elbow	Forearm	2.03	2.97	13.8	Elbow – Forearm	120	59

Evidence of ongoing denervation with reinnervation was seen in the left upper trunk-supplied muscles. Of note, an MRI of the brachial plexus and brain with and without contrast did not reveal any abnormalities. Lyme antibody (Ab) in serum was negative. The patient was diagnosed with VZV plexopathy. His symptoms improved with physical therapy, and the pain was moderately controlled with pregabalin. Follow-up 18 months after the initial diagnosis revealed residual pain in the left upper extremity that is not functionally limiting and managed with only acetaminophen as needed.

## Discussion

This study describes the case of a patient with oligodendroglioma who had undergone resections, chemotherapy, and radiotherapy, and later developed brachial plexopathy following a VZV infection. Interestingly, our patient was rather young, at 44 years old. Prior studies have described VZV-associated plexopathy in mainly patients over 60 years old. Furthermore, his clinical condition was explored for several months, and priority was given to investigating possible compression injuries. Given his extensive history with cancer and subsequent treatments, VZV should have been included in the differential earlier in his course.

Additionally, MRI findings may support the diagnosis of VZV-associated plexopathy, but such was not the case in our patient. A case series looked at 10 patients and found MR to be diagnostic for plexopathies in 70% of patients, as defined by T2 hyperintensity and other secondary findings such as thickening of the nerve roots [8,9]. Of note, contrast enhancement was not found in any of the cases described by the case series [8]. MR imaging does prove crucial in excluding other pathologies, such as spinal cord compression, but it may not demonstrate T2 hyperintensity or contrast enhancement as may be expected in cases of plexopathy. Also, if the MRI is not available, electrodiagnostic studies combined with the clinical presentation and history of painful blistering rash should be diagnostic for VZV plexopathy. There are case reports that showed positive VZV DNA polymerase chain reaction (PCR) in cerebrospinal fluid (CSF) even four weeks after the initial blistering rash [4]. Also of diagnostic importance, the presence of anti-VZV immunoglobulin (Ig) G antibody in the CSF is more sensitive than VZV DNA [10].

## Conclusions

Although rare, VZV-associated plexopathy should be considered among the differential diagnosis in patients who present with plexopathy or radiculopathy after a recent HZ diagnosis. Although excluding structural causes is important, delay in the diagnosis may negatively affect the prognosis and may create more and longer-term disability for patients. Despite mostly affecting elderly patients, younger patients with an impaired immune system may be affected as well. Timely initiation of acyclovir treatment and physical therapy may improve the patient’s outcomes.
